# Retraction: Effectiveness of carbon dioxide cryotherapy for the treatment of cutaneous leishmaniasis: Systematic review and meta-analysis

**DOI:** 10.1371/journal.pntd.0014426

**Published:** 2026-06-16

**Authors:** The PLOS Neglected Tropical Diseases Editors

After this article [1] was published, concerns were raised about the reported study, and corresponding author FTZ contacted *PLOS Neglected Tropical Diseases* to notify the journal of a discrepancy between the reported focus of the article and the studies included in the systematic review and meta-analysis.

The following issues were noted in editorial follow-up:

- The stated aim for the study is “to summarize the pooled evidence on the effectiveness of carbon dioxide-based cryotherapy for the treatment of cutaneous leishmaniasis”; however, most (but not all) included studies appear to evaluate a different treatment, carbon dioxide laser therapy.
- Inaccuracies in the referencing of included studies that limits support for, and independent evaluation of, the systematic review and meta-analysis outcomes:
  ◦ There appear to be discrepancies between reference numbering used in S4 Table of [1] and in the Reference list of [1].
  ◦ Descriptions of some of the studies in the Characteristics of included primary studies subsection of the Results section do not appear to fully align with the specific in-text citations provided.
  ◦ The Reference list for [1] does not appear to include a study fully matching the description of Nilforoushzadeh MA et al. (2014) reported in S4 Table.
  ◦ Tilahun F et al. (2023) is reported in S4 Table and included in both the systematic review and meta-analysis, but details of the associated study are not sufficiently reported to allow readers to access the study data.
- Further concerns about methodology and analyses:
  ◦ The search strategy may not be sufficiently comprehensive.
    - The use of a free-full-text filter may restrict retrieval.
    - No supplementary search methods are reported, such as backward citation tracking or cited-by searches.
  ◦ The selection and application of statistical methodology is unclear. In Figs 4-6, values labeled as “effect sizes” range from 64.0 to 95.5. These values appear to represent cure rates (percentages) with reported 95% confidence intervals extending below 0% and/or above 100%.
  ◦ In S3 Table, some overall bias ratings in the risk-of-bias assessment appear to be inconsistent with the domain level assessments.
  ◦ The article [1] does not include an assessment of the certainty of evidence, for example using the GRADE framework, limiting interpretation of the strength and reliability of the reported findings.
- The published link to the underlying study data for [1] is not functional.

The authors did not respond to editorial requests for comment on the above-mentioned concerns.

In light of these concerns, which call into question the validity and reliability of the published results and conclusions, the *PLOS Neglected Tropical Diseases* Editors retract this article [1].

EGB agreed with the retraction. FTZ did not agree with the retraction. BMG and KAG either did not respond directly or could not be reached.

## References

[1] Tilahun Zewdu F, Misganaw Geremew B, Gadisa Belachew E, Alemu Gelay K. RETRACTED: Effectiveness of carbon dioxide cryotherapy for the treatment of cutaneous leishmaniasis: Systematic review and meta-analysis. PLoS Negl Trop Dis. 2025;19(1):e0012741. doi: 10.1371/journal.pntd.0012741 39761302 PMC11771858

